# Tuning Polyphenols for Thermosetting Wood Adhesives: Class ‘A1’ Non-Isocyanate Polyurethanes from Condensed Tannins

**DOI:** 10.1021/acs.jafc.5c02453

**Published:** 2025-06-27

**Authors:** Gopakumar Sivasankarapillai, Arsène Bikoro Bi Athomo, Detlef Schmiedl, Antonio Pizzi, Marie-Pierre Laborie

**Affiliations:** † Freiburg Materials Research Centre, 9174 University of Freiburg, Stefan-Meier-Strasse 21, D-79104 Freiburg i. Br., Germany; ‡ Chair of Forest Biomaterials, Faculty of Environment and Natural Resources, University of Freiburg, Werthmannstrasse 6, D-79085 Freiburg i. Br., Germany; § Department of Environmental Engineering, Fraunhofer Institute for Chemical Technology ICT, Joseph-Von-Fraunhofer-Str. 7, 76327 Pfinztal, Germany; ∥ Lermab-Enstib, University of Lorraine, 27 rue Philippe Seguin, 88000 Epinal, France; ⊥ Institut Charles Sadron, 23 rue du Loess, BP 84047, 67034 Strasbourg Cedex 2, France

**Keywords:** condensed tannins, nonisocyanate polyurethane, derivatization, polycondensation, shear bond strength, adhesive class

## Abstract

This study aimed to develop nonisocyanate polyurethane (NIPU) wood adhesives from Quebracho condensed tannins (CT). Following the carbonation of CTs with dimethyl carbonate, condensation with multifunctional amines delivered two distinct NIPU formulations: TANIPU-DA when hexamethylene diamine (DA) was used and TANIPU-DA-TD when successively using DA and hexamethylenetetramine (TD). For both TANIPUs, urethane formation was confirmed with Fourier-transform infrared (FTIR), viscosity measurements, and size exclusion chromatography (SEC). Gelation and vitrification were evidenced with DMA in the 120–150 °C and the 160–220 °C ranges, respectively. TANIPU-DA-TD wood-bonded assemblies reached a lap shear strength exceeding 10 N/mm^2^, qualifying TANIPU-DA-TD as a class ‘A1’ wood adhesive according to European standards. TANIPU-DA and TANIPU-DA-TD achieved relatively high wet strengths of 2.5 ± 1.3 and 3.8 ± 1.2 N/mm^2^, respectively. This study demonstrates that tannin-based NIPUs can meet the dry bonding strength requirements for wood adhesives, suggesting a viable, more environmentally friendly alternative to traditional formaldehyde- and isocyanate-based wood adhesives.

## Graphical abstract

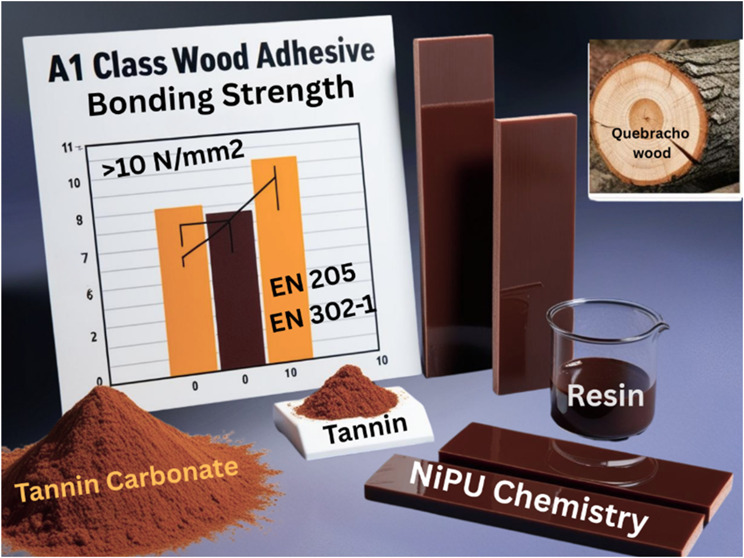

## 1 Introduction

The Wood adhesives and binders market continues to grow, and its value is expected to reach 21.9 US Billion by 2028.^1^ Although it is still dominated by synthetic formaldehyde and isocyanate-based adhesive resins, the wood adhesive industry has long been striving to offer more environmentally and health-friendly binders.^2,3^ Indeed, increasingly stringent environmental regulations on VOCs prompt replacing “substances of very deep concern”, such as formaldehyde- and isocyanate-based compounds. Albeit wood itself emits formaldehyde,^4^ formaldehyde is particularly subject to strict restrictions in wood products, as it is classified as “carcinogenic for humans”.^2,5^ Increasing public awareness calls for biobased wood adhesives free of formaldehyde, isocyanates and other VOCs. Academics and industries have thus heavily engaged in developing VOC-free wood adhesives sourced from renewables. Several companies are making significant advancements in this direction. Companies such as BindEthics, Covestro (Desmopan EC), and Henkel (Loctite HB S ECO & CR821 ECO) are pioneering advancements in biobased polyurethane adhesives.^6,7^ Numerous biobased systems have also been explored as potential wood adhesives in academic research, and the readers are referred to excellent research reviews on this topic.^8–11^ Most notably, Li’s group made significant advances in wood adhesives that were concomitantly VOC-free and biobased in the early 2000s by reacting condensed tannins with polyethylenimine.^12^ The resulting adhesive exhibited dry shear strength approaching the required value of 10 N/mm^2^ for wood bonding in interior applications and particularly stood out for its water resistance. Soy proteins have been reacted with aliphatic polyketones to produce VOC-free wood adhesives with moderate shear strength (3 N/mm^2^ max.).^13^ Solutions of dialdehyde cellulose have also proven to be promising wood adhesives^14^ delivering a lap shear strength on beech veneers of 9.6 N/mm^2^. Nonisocyanate polyurethanes (NIPUs) from biobased macromonomers such as lignin and condensed tannins have similarly been the topic of extensive research.^11,15–20^ Among the various methods for synthesizing nonisocyanate polyurethanes (NIPUs), the condensation of polyphenol carbonates with multifunctional amines has demonstrated significant potential. This is particularly true for condensed tannins, which have shown greater reactivity and better wood bonding capabilities than lignin.^21,22^ Transesterification of tannins with dimethyl carbonate (DMC) followed by polycondensation with hexamethylenediamine (DA) has been demonstrated.^23^ Alternatively, the coreaction of an aminated tannin with carbonated tannins also delivers NIPUs.^24^ From the sustainability standpoint, a cradle-to-grave life cycle analysis has revealed environmental advantages of NIPUs from tannin carbonates over other tannin-based polymeric adhesives and their synthetic amino plastic counterparts.^25,26^ However, bioadhesives must deliver a bonding performance similar to synthetic adhesives to be a feasible alternative. For indoor structural applications, wood adhesives must meet the bonding performance requirements of the European Standard test EN 302-Part 1 (2013). To the best of our knowledge, no reports of tannin-based, formaldehyde- and isocyanate-free wood adhesives pass the Class ‘A1’ bond shear strength requirement. The research aims to extend the development of biobased NIPU wood adhesives based on an industrial quebracho tannin extract soluble fraction to reach adhesive bonding strength requirements for structural applications.

## 2 Experimental Section

### 2.1 Materials

Quebracho tannin extracted from the wood of was supplied by Silva Chimica (St. Michele Mondovi, Italy). Hexamethylenediamine (DA 98%), (DMC, 99%, anhydrous), hexamethylenetetramine (TD, 99%, ACS reagent, 100–97–0) and “DBU” (1,8-diazabicyclo[5.4.0]­undec-7-ene), sodium hydroxide (NaOH), (Analytical reagent) (98.87%), pyridine (ACS reagent 99% and anhydrous 99.8%), deuterated chloroform (CDCl_3_), anhydrous *N*,*N*-dimethylformamide (*N*,*N*-DMF), Phosphytilating reagent (2-chloro-4,4,5,5-tetramethyl-1,3,2-dioxaphospholate, TMDP), cholesterol (57–88–5, 99%), chromium acetylacetonate (21679–31–2), acetic anhydride, were supplied by Sigma-Aldrich (Taufkirchen, Germany). All chemical reagents were used without further purification.

### 2.2 Methods

#### 2.2.1 Carbonation of Quebracho Tannin Extract Soluble Fraction (QSF)

Carbonated tannins were synthesized by transesterifying the soluble fraction of quebracho tannin extract (QSF) with dimethyl carbonate (DMC) in an aqueous medium. The process started by mixing approximately 10 g of vacuum-dried QSF with 11 g of DMC into a double-neck round-bottom flask equipped with nitrogen flow and a reflux condenser. Next, 0.185 g of 1,8-diazabicyclo [5.4.0] undec-7-ene (DBU) catalyst in 12.5 g of deionized water was added to the flask. The reaction was conducted for 6 h at room temperature (20 ± 2) °C in a nitrogen atmosphere. After completion, water and excess DMC were removed by using a rotary evaporator. The wet product was collected and vacuum-dried at 40 °C and 70 mbar for 48 h. The reaction was further scaled up, yielding approximately 40 g of QSF. The dried carbonated tannin (QSF-Cb) was analyzed using Fourier-transform infrared (FTIR), size exclusion chromatography (SEC), 2D NMR (HSQC), ^31^P NMR, and TGA/MS. The carbonation degree was calculated from the difference in phenolic OH contents of QSF before and after carbonation, as measured from ^31^P NMR on QSF derivatives after in situ labeling with TMDP, respectively.

#### 2.2.2 Preparation of Stage B Tannin-NIPU Resins

Two Tannin-NIPU (TANIPU) resins were synthesized utilizing two distinct cross-linking methodologies. The initial method employed hexamethylene diamine (DA) as the exclusive cross-linker, resulting in a stage B resin with a targeted solid content of 40%, designated as TANIPU-DA. The formulation of the second resin sought to achieve a targeted solid content of 50%. This process mirrored the synthesis of TANIPU-DA for five min prior to the termination of the cooking process. At that juncture, hexamethylenetetramine (TD) was introduced as an additional cross-linker into the reaction vessel, yielding a stage B resin subsequently referred to as TANIPU-DA-TD. The resin cooks proceeded as follows: QSF-Cb and water were mixed into a reaction flask equipped with a reflux condenser, after which DA was added to reach a carbonate/amine (Cb/NH_2_) functional groups molar ratio of 1:1.77 based on the carbonate content assessed from ^31^P NMR data. The reaction temperature was raised to (55 ± 2) °C and occasionally stirred for approximately 30 min before adding 0.523 mol of NaOH per mol of QSF-Cb phenolic hydroxyls as determined from ^31^P NMR data. Thereafter, the temperature was increased to 90 °C, and the cook proceeded under magnetic stirring for 90 min before quenching with liquid nitrogen. For TANIPU-DA-TD, 10 mg (0.071 mmol) of TD per gram of QSF-Cb was added 5 min before stopping the cooking process. The resins were immediately portioned into small containers, quenched with liquid nitrogen, and stored in a freezer until further use. Note that residual DBU from the carbonation reaction might have been present in the reaction kettle. Scheme 1 illustrates the expected synthetic pathway for both of the TANIPU resins. In Stage A, DMC reacts with the hydroxyl groups of the QSF tannin to form carbonated tannins (QSF-Cb). In Stage B, DA reacts with the carbonate groups of QSF-Cb to form urethane linkages in both TANIPU-DA and TANIPU-DA-TD. Additional cross-linking chemistry is expected in TANIPU-DA-TD due to the nucleophilic attack of reactive imino-amino methylene bases generated through TD decomposition. Indeed, under alkaline conditions (pH 11–12 in this work) and in the presence of condensed tannins, it has been established that TD preferentially decomposes into imine products rather than formaldehyde, rapidly attacking the electronegative ortho and para positions in the phenolic rings.^27–30^ During the curing process of the resin, it is expected that the reactions driven by the terminal amine of the stage B TANIPU oligomer, alongside unreacted DA and residual hydroxyl groups from the QSF-Cb, as well as those resulting from the imine carbocations formed from any undecomposed TD, will lead to the creation of a cross-linked stage-C resin.^31,32^

**Scheme 1:**
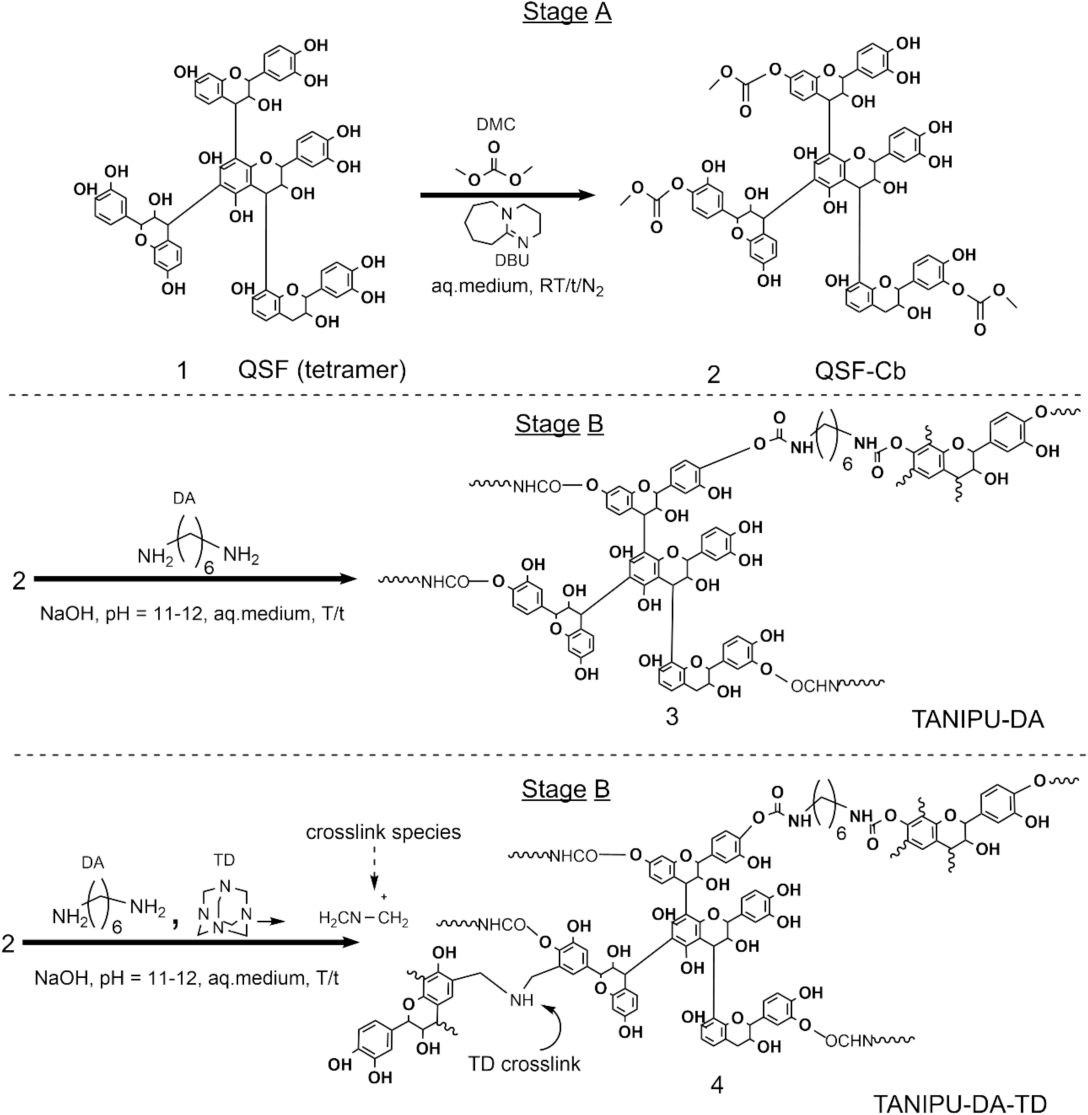
Synthetic Pathway for the Transesterification of QSF with DMC (QSF-Cb) in Stage A Resin, and the Carbamation Reaction of QSF-Cb with DA (TANIPU-DA) and Nucleophilic Crosslinking of TANIPU-DA with TD (TANIPU-DA-TD) in Stage B Resin

The cross-linker DA was selected due to its medium-length alkyl chain, which contributes to enhanced molecular flexibility and improved interfacial bonding performance. It also offers lower toxicity and greater safety than aromatic or branched alternatives, along with favorable commercial availability for industrial application. To further enhance bonding performance, TD was introduced as a secondary cross-linker, leveraging the high reactivity of its decomposition intermediates toward nucleophilic sites within the flavonoid structures of tannin, thereby reinforcing the network architecture of TANIPU-DA.

#### 2.2.3 Physico-Chemical Characterization of Stage B-TANIPU Resins

The relative viscosity of TANIPU resins was measured at ambient temperature (22 ± 2) °C using a Vibro-Viscosimeter SV-10 (0.3–10, 000 mPa.s). The solid content was determined gravimetrically through freeze-drying with an α 1–2 LD Plus freeze-dryer operating at −53 °C/0.69 mbar for 48 h.

#### 2.2.4 Fourier-Transform Infrared Spectroscopy (FTIR)

The FTIR analysis was used to investigate the chemical composition of dried QSF, QSF-Cb, and stage B-TANIPU resins. A PerkinElmer FTIR spectrometer model 65 was used in attenuated total reflection (ATR) mode with a ZnSe crystal, acquiring 32 scans over a spectral range of 4000 to 650 cm^–1^ at a resolution of 4 cm^–1^. The spectra of the TANIPU resins were normalized to the C–H vibrations of the aromatic ring at 1513 cm^–1^ to account for variations in sample characteristics.

#### 2.2.5 Size Exclusion Chromatography (SEC)

Number and weight-average molar weights (*M_n_* and *M*_w_) were determined by size exclusion chromatography (SEC) on acetylated QSF and TANIPU-DA. For acetylation, approximately 100 mg of vacuum-dried QSF and TANIPU-DA were mixed with 2 mL each of acetic anhydride: pyridine (1:1, v/v) solvent mixture and stirred in a closed round-bottom flask at ambient temperature for 48 h.^33^ Then, 25 mL of ethanol was added and evaporated with a rotary evaporator to remove excess acetic anhydride and pyridine. This washing step was repeated five times. The acetylated derivative was dried at 40 °C for 24 h at 70 mbar. The molecular weights of the QSF and TANIPU-DA resins were determined on a GPC SECurity 1200 system (PSS-Polymer Standards Service). The system used dimethylacetamide (DMAc) with 0.5% lithium bromide as the eluent. The temperature was set to 25 °C and equipped with GRAM columns, 30 to 10,000 Å and a refractive index (RI) detector. The GPC system was calibrated using poly­(methyl methacrylate) (PMMA) standards.

#### 2.2.6 Matrix-Assisted Laser Desorption Ionization Time-of-Flight (MALDI-TOF): Mass Spectrometry Analysis of QSF

The samples were dissolved in a solution of water/acetone (1:1, v/v). Up to 7.5 mg/mL NaCl solution (1.5 μL of 0.1 M) in a methanol/water mixture (1:1) was added and placed on the MALDI target to increase ion formation. The solutions of the sample and the matrix were mixed in equal amounts, and 1.5 μL of the resulting solution was placed on the MALDI target. A matrix of 2,5-dihydroxy benzoic acid was used. Red phosphorus (500–3000 Da) was used as a reference for spectrum calibration. Finally, the MALDI target was introduced into the spectrometer after the evaporation of the solvent. The spectra were recorded on a KRATOS AXIMA Performance mass spectrometer from Shimadzu Biotech (Kratos Analytical Shimadzu Europe Ltd., Manchester, U.K.). The irradiation source was a pulsed nitrogen laser with a wavelength of 337 nm. The length of one laser pulse was three ns. Measurements were carried out using the following conditions: polarity-positive, flight path-linear, 20 kV acceleration voltages, and 100–150 pulses per spectrum. The delayed extraction technique was used, applying delay times of 200–800 ns. The software MALDI-MS was used for the data treatment.^34^

#### 2.2.7 2D-NMR, HSQC of QSF and QSF-Cb

^1^H–^13^C HSQC NMR spectra were recorded at 25 °C using a Bruker Biospin Avance Neo (^1^H 500.13 MHz, 13C 125.76 MHz) instrument with TopSpin 4.1 software and a 5 mm 1H/BBF probe featuring z-gradients. The spectra were captured in DMSO-*d*_6_ or *N*,*N*-DMF-*d*_7_ and referenced to the residual signals: 2.49 ppm (^1^H) and 39.5 ppm (^13^C) for DMSO-*d*_6_, and 2.92 ppm (^1^H) and 34.89 ppm (^13^C) for N, N-DMF-*d*_7_. The ^13^C NMR spectra were collected by using 16K scans, requiring 7 h and 20 min of acquisition time. For the ^1^H–^13^C HSQC experiments, 64 scans were acquired using the standard Bruker pulse program (hsqcetgp) with the following parameters: Acquisition: TD 2048 (F2), 256 (F1); SW 16.4 ppm (F2), 165 ppm (F1); O1 3088.43 Hz; O2 9432.21 Hz; D1 = 1.60 s; CNST2 = 145. The acquisition time was 62 ms for the F2 channel and 6.17 ms for the F1 channel. Processing parameters include SI = 2048 (F2, F1), WDW = QSINE, LB = 1.00 Hz (F2), and 0.30 Hz (F1); PH_mod = pk;Baseline correction ABSG = 5 (F2, F1), BCFW = 1.00 ppm, BCmod = quad (F2), and no (F1); Linear prediction = no (F2), LPfr (F1). A sample size of 50 mg (vacuum-dried at 50 °C for 96 h under 7 mbar) was used in 600 μL of DMSO-*d*_6_ or 600 μL of *N*,*N*-DMF-*d*_7_. Both NMR solvents underwent independent analyses to evaluate any potential solvent effects on the presence of cross-coupling signals (stability test) and the solubility of the samples. No solvent effect was observed.

#### 2.2.8 ^31^P NMR of QSF and QSF-Cb

^31^P NMR spectra of TMDP-in situ-labeled QSF and QSF-Cb were recorded on a 300 MHz Avance 300 with an interpulse delay of 10 s (128 scans). For Phosphytilation, 20–25 mg QSF was dissolved in anhydrous pyridine and deuterated chloroform (500 μL, 1.6:1, v/v) as per Meng et al.,^35^ and 0.75 μL of anhydrous dimethylformamide. Subsequently, the internal standard solution (100 μL, 1 mmol of cholesterol in 10 mL of the pyridine/CDCl_3_ solvent mixture) and the relaxation reagent (chromium acetylacetonate (III), 25 μL, 0.0312 mol L^–1^) were added. All mixtures were then reacted with 100 μL of a phosphotilating reagent (2-chloro-4,4,5,5-tetramethyl-1,3,2-dioxaphospholate, TMDP) for 10 min and transferred into 5 mm NMR tubes. The chemical shifts relative to the reaction product of TMDP with water at 132.2 ppm were used to calibrate the NMR signals and assigned the signal range at δ = 150.0–145.5 ppm for aliphatic −OH groups, 145.5–144.7 ppm for cholesterol (internal standard), 144.7–136.6 ppm, and 136.6–133.6 ppm for phenolic −OH and carboxylic acids groups, respectively.

#### 2.2.9 Thermal Stability and Cure Behavior of Stage B TANIPU Resins

(a) Thermogravimetric Analysis (TGA): Approx. 5 mg of TANIPU resin was heated at 700 °C with a rate of 10 °C/min under a nitrogen atmosphere, using a Pyris 1 Thermogravimetric Analyzer (PerkinElmer). This TG analyzer assessed the thermal stability and water content of the TANIPU resins.
(b) Dynamic Mechanical Analysis (DMA): The dynamic mechanical analyzer (PerkinElmer, Model DMA 8000) was used to evaluate the curing behavior of the TANIPU resins. Approx. 30 mg of liquid resin was deposited into a stainless-steel pocket and allowed to air-dry at ambient temperature for 30 min. The sample viscoelastic properties were measured in single cantilever beam mode at 1 Hz during a heating scan from 25 to 250 °C at 5 °C/min.

#### 2.2.10 Physico-Mechanical Characterization of Stage C TANIPU Resins

According to the EN 205 (2003) standard, two straight-grained beechwood panels were selected and cut across the grain to approximately 200 mm × 130 mm, ensuring uniformity and the absence of defects. The thickness of the wood panel was maintained at (5.0 ± 0.1) mm. Approx. 150 g/m^2^ of the solid content of stage B TANIPU adhesive was uniformly applied to the bonding surfaces of two additional beechwood panels (approximately 200 mm × 57 mm × 5 mm). The adhesive-coated panels were then hot-pressed at 200 °C for 600 s. under a pressure of 1 N/mm^2^ using a hydraulic hot press (LaboPress P 200 S, Vogt Maschinenbau GmbH). From these bonded assemblies, slotted specimens were cut to dimensions of (150 ± 5) mm in length and (20.0 ± 0.2) mm in width, with an overlap length (bond line) of (10.0 ± 0.2) mm. A total of 8–12 shear test samples were prepared for each adhesive formulation. All specimens were then conditioned at a controlled temperature of 20 °C and 65% relative humidity for 7 days before testing.

Mechanical testing followed the guidelines of EN 205 (2003) and DIN EN 302–1 (2013). Specimens were tested using a Zwick/Roell Z100 universal testing machine (Macrosense, Zwick/Roell, Ulm, Germany). They were tested in load mode at 2 kN/min to ensure controlled loading conditions, and the maximum load at failure was recorded. Shear strength was calculated by dividing the load by the bonded area (20 mm × 10 mm = 200 mm^2^). Postfailure, the fracture surfaces were visually inspected to estimate the percentage of cohesive wood failure, providing insight into adhesive performance.

## 3 Results and Discussion

The analysis of QSF tannin using MALDI-TOF revealed the presence of various condensed forms of catechin (*m*/*z* 290) and fisetinidin (*m*/*z* 274) units. Dimers (*m*/*z* 600–665) and tetramers (*m*/*z* 1086, 1130) appeared in high intensity, whereas trimers (*m*/*z* 857, 873) and pentamers (1402, 1419) appeared in low intensity peaks (Figure S1 and Scheme 2). These findings are consistent with existing literature.^34,36^

**Scheme 2:**
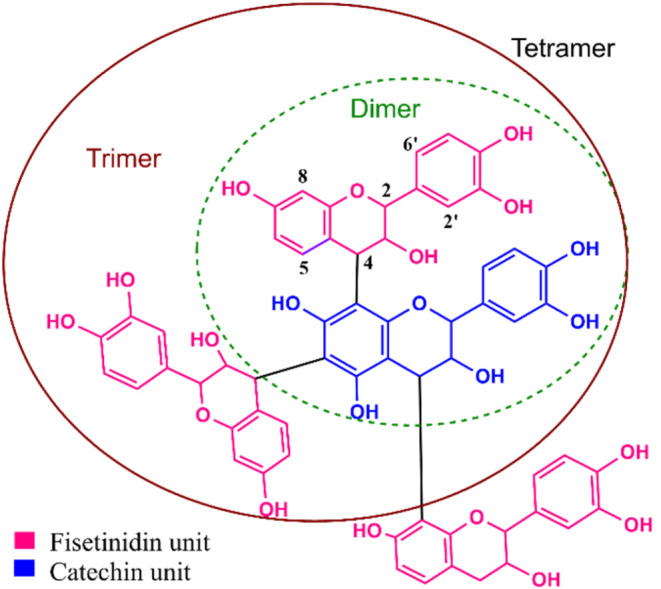
Representative Structure of Quebracho Proanthocyanidins Comprising Catechin as the Starting Unit and Fisetinidin as the Extender Units

### 3.1 Molecular Structure of QSF and QSF-Cb

The QSF and QSF-Cb were analyzed using 2D NMR and ^31^P NMR techniques. The 2D NMR (HSQC, 500 MHz) indicates cross-coupling signals located at δH: 3–5/δC: 52–95 ppm, corresponding to the aliphatic carbons and protons related to the C-ring and carbohydrates. (Figure 1). This region shows significant signals due to the partial overlap of aliphatic protons and carbon signals from the C-ring and carbohydrate impurities.^37^ The spectra of QSF-Cb indicate the presence of carbonate groups (Cb-group), with a cross-coupling signal at δH: 3.46/δC: 48.10 ppm, corresponding to H_3_C–O–COO-phenyl in the aliphatic region (Figure 1). In the aromatic region of the spectra (Figure 1), cross-coupling signals for the C6–H6 (δH: 6–6.6 ppm/δC: 100–105 ppm) and C8–H8 at δH: 6–6.6 ppm/δC: 105–110 ppm within ring A are noted. Furthermore, a cross-coupling signal for the C5–H5 (δH: 6.4–7.0 ppm/δC:127–132 ppm) indicates that no condensation is associated with this position in ring A and no other bond than C5–H5 is present there. If position C5 in the A-ring is occupied by, e.g., OH-groups instead of H-Atom, then a cross-coupling signal δ^1^H/ δ^13^C will not be observed. The signals in the range of δH: 6.2–7.7 ppm are linked to the aromatic B-ring cross-coupling signals of QSF, appearing around δC: 115–122 ppm, specifically corresponding to positions 2′, 5′, and 6′ in the ring B.^33,35^ No distortion in the chemical shifts occurs upon derivatization except for a minor variation in ^13^C NMR signals of QSF-Cb at the aliphatic region associated with the C-ring and carbohydrates.^39^Overall, the analysis of the 2D NMR spectra of QSF and QSF-Cb illustrates the presence of a fisetinidin subunit in tannin QSF, which is parallel to the finding from MALDI-TOF analysis and also confirms the existence of carbonate groups in QSF-Cb.

**Figure 1:**
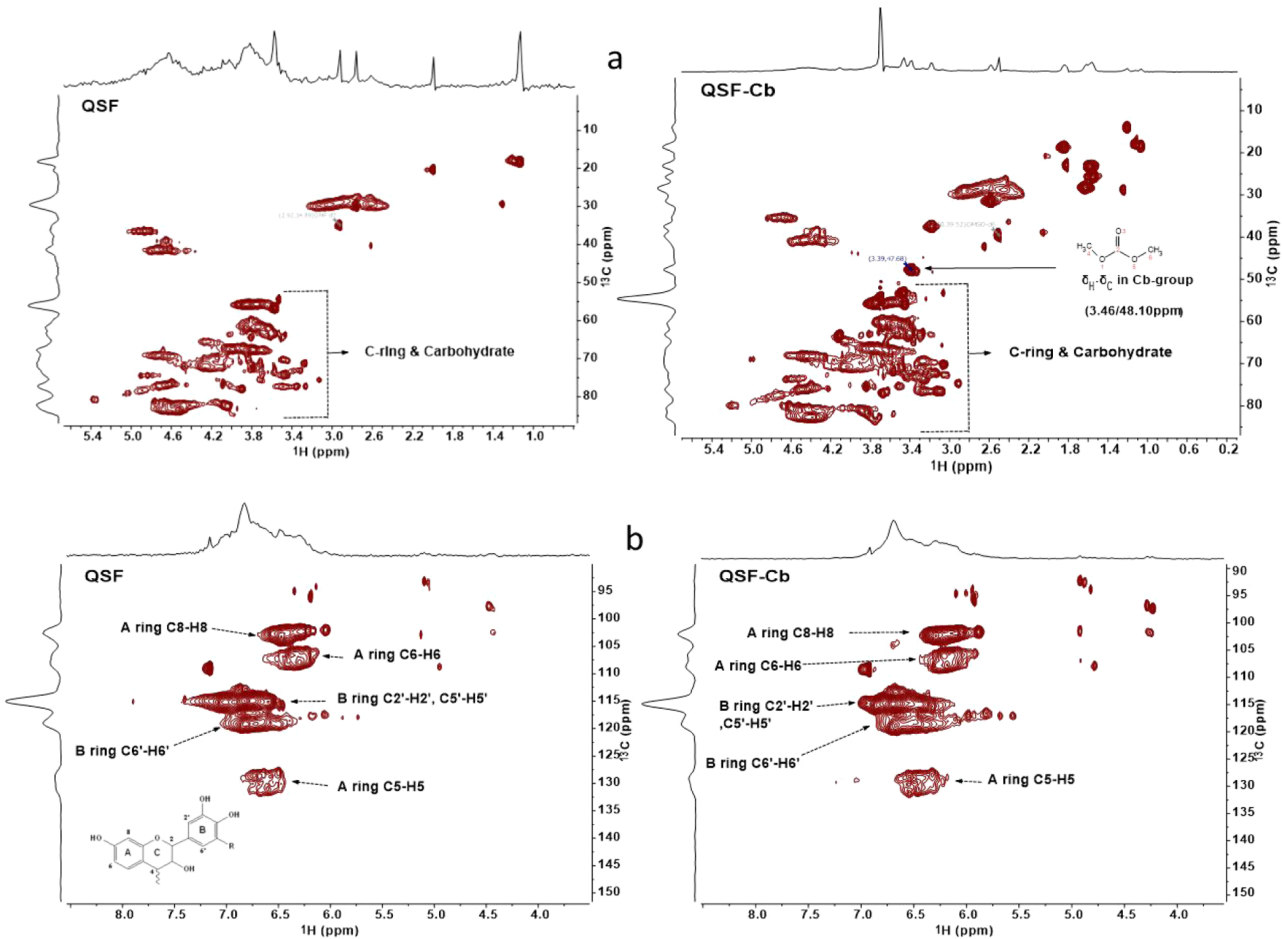
2D NMR-HSQC spectra of QSF and QSF-Cb with a focus on the aliphatic region (a) and aromatic region (b), revealing carbonation in stage A resin.

The ^31^P NMR spectra of phosphytilated QSF and QSF-Cb exhibit aliphatic and aromatic OH group resonances at 145–149 ppm and in the 136–141 ppm regions, respectively. Carboxylic acids from gallic acids also appear around 135 ppm (Figure 2).^37^ Notably, two resonances in the aromatic OH region centered around 139 and 137.5 ppm can be ascribed to OH groups of the B and A rings of the QSF.^37^ The aliphatic region in the ^31^P NMR spectrum reveals aliphatic OH from the C-ring and glycosylated tannins impurities.^37^ As these signals overlap, the aliphatic OH region was not considered for quantification and the DS was tentatively estimated from the integration of aromatic OH resonances only (Table 1). Note, finally, the absence of pyrogallol hydroxyl signals in the 142.5–141.8 ppm range, despite evidence in the MALDI-TOF analysis of small quantities of protonated gallocatechin fractions. (*m*/*z*,308). In QSF, the total content of aromatic OH is 9.47 mmol/g, distributed between the A and B rings in a 30:70 ratio (Table 1). This ratio is consistent with prior reports of the chemical nature of quebracho proanthocyanidins, which essentially comprise catechin starting units and fisetinidin extender units.^36^ Upon carbonation, the phenolic OH content in QSF decreases, confirming that transesterification with DMC has occurred in the aromatic OH groups. The integration of the OH aromatic signals during carbonation indicates a loss of approximately 29%, corresponding to the total degree of substitution (Table 1). Carbonation primarily occurs within the A-ring of the QSF and to a considerably lesser extent within the B-ring (Table 1). Additionally, there is a notable rise in the aliphatic OH region after carbonation. We attribute this to hydrolysis from the glycosylated tannins, as QSF-Cb exhibits greater solubility in polar solvents, and the phosphytilating agents are particularly reactive.

**Figure 2:**
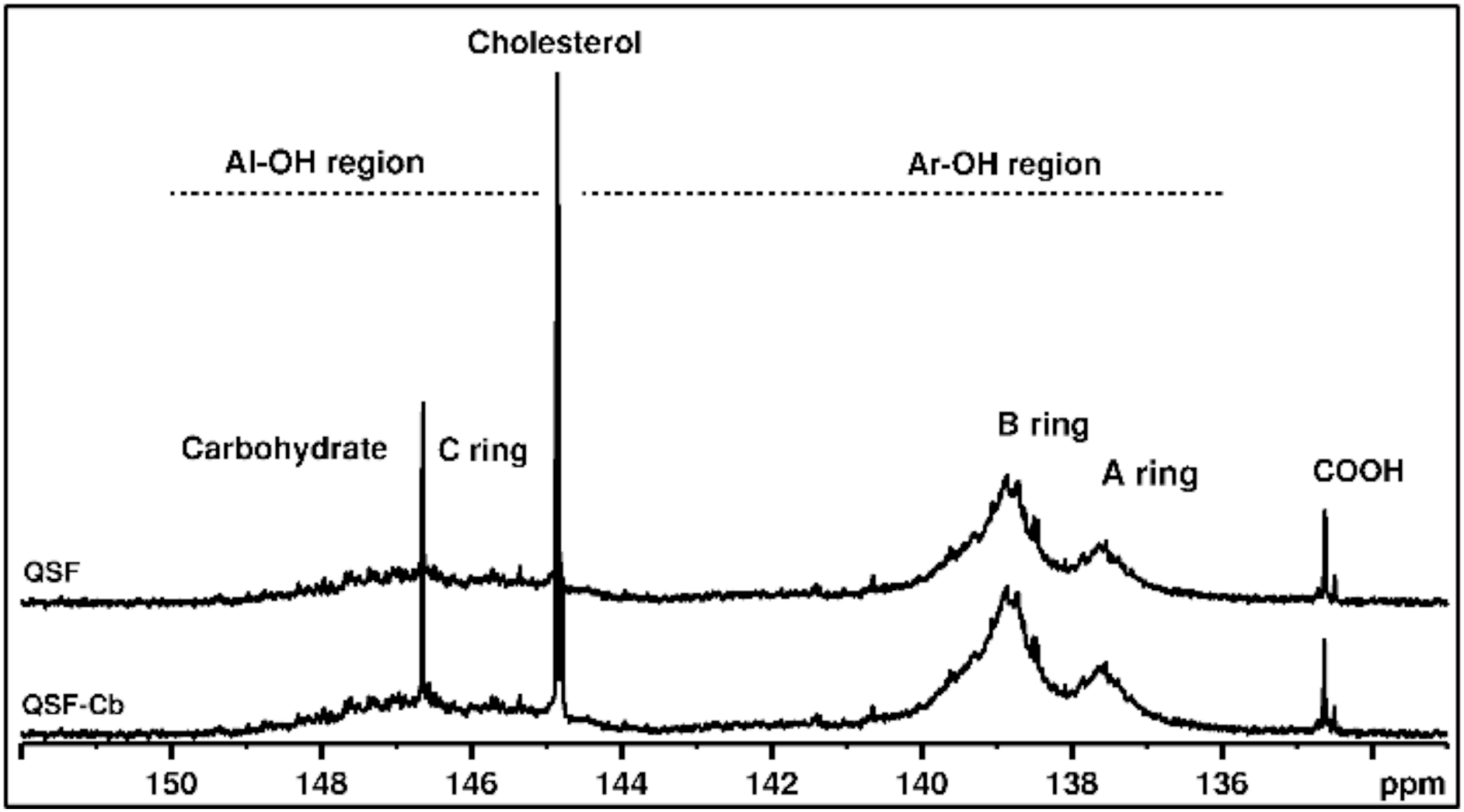
^31^P NMR spectra of *in situ*-labeled QSF and QSF-Cb enabling the determination of the degree of substitution in carbonate groups in QSF-Cb from the aromatic OH contents using cholesterol as an internal standard.

**Table 1:** Functional Group Analysis of QSF and QSF-Cb Based on ^31^P NMR Data

sample	A-ring phenolic OH	B-ring phenolic OH	total phenolic OH	C-ring and carbohydrates aliphatic OH
(mmol/g)				
QSF	2.38	7.08	9.47	1.92
QSF-Cb	0.54	6.15	6.69	2.74^a^
Degree of Carbonation of QSF (DS)				
	77%	13%	29%	

a Increase upon carbonation is ascribed to hydrolysis of glycosylated tannins and is not representative of the content of aliphatic OH on the C ring of QSF-Cb.

### 3.2 Physicochemical Properties of Stage B TANIPU Resins

The FTIR spectroscopy was used to monitor the sequential reactions from QSF (Figure 3a,b). The band at 3300 cm^–1^ corresponds to the stretching vibrations of the hydroxyl groups, while the aromatic ring’s characteristic stretching vibrations occur at 1608 and 1512 cm^–1^. In QSF, the broad stretching vibration of the OH groups around 3300 cm^–1^ decreases upon carbonation, whereas a carbonyl stretching vibration emerges at 1740–1720 cm^–1^. This signal likely originates from the carbonate groups in QSF-Cb.^38^ The disappearance of these carbonyl stretching vibrations upon condensation with DA confirms their consumption. In the TANIPU resins, stretching vibrations from the C=O and N–C bonds are identified at 1713 and 1588 cm^–1^, respectively, indicating the presence of urethane bonds. Additionally, the bands at 2940 and 2840 cm^–1^ represent the alkyl groups from DA and TD.^39^ The bands around 1600 cm^–1^ are broadened primarily between 1680 and 1540 cm^–1^ in TANIPU-DA, and this broadening is even more pronounced in TANIPU-DA-TD. Additionally, bands appearing at 1266 cm^–1^ in TANIPU-DA and at 1259 cm^–1^ in TANIPU-DA-TD indicate amine C–N elongation, providing further evidence of urethane linkages in the TANIPU resins.^24,40–42^

**Figure 3:**
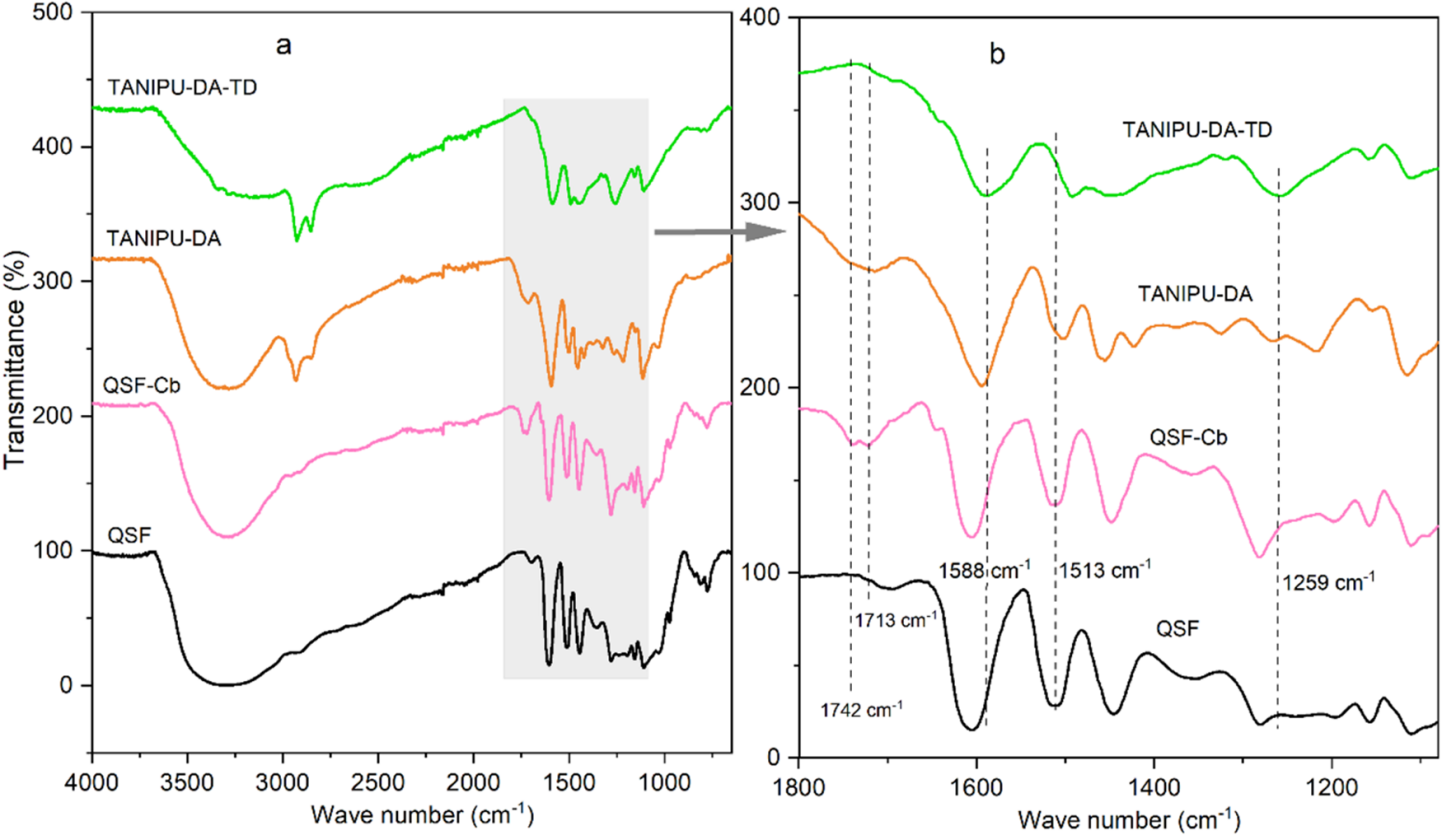
Normalized FTIR spectra of QSF, QSF-Cb and TANIPU resins (a); and their fingerprint region (b), revealing carbonation in stage A resin and urethane linkage formation in stage B resin.

Further evidence of the efficient condensation of QSF tannin is obtained by comparing the molecular weight profiles of neat QSF and TANIPU-DA. The chromatogram for TANIPU-DA confirms an increase in molecular weight, and it reveals a broad molecular weight distribution with a polydispersity of 3.84, even though the resin underwent acetylation before SEC measurement, which also affects SEC measurements (Figure 4).^43,44^ Although SEC analysis was not performed on TANIPU-DA-TD, it is noteworthy that the viscosity substantially increases from 995 ± 20 mPa·s for TANIPU-DA to 2000 ± 50 mPa·s for TANIPU-DA-TD, suggesting an even higher molecular weight or higher branching for the latter.

**Figure 4:**
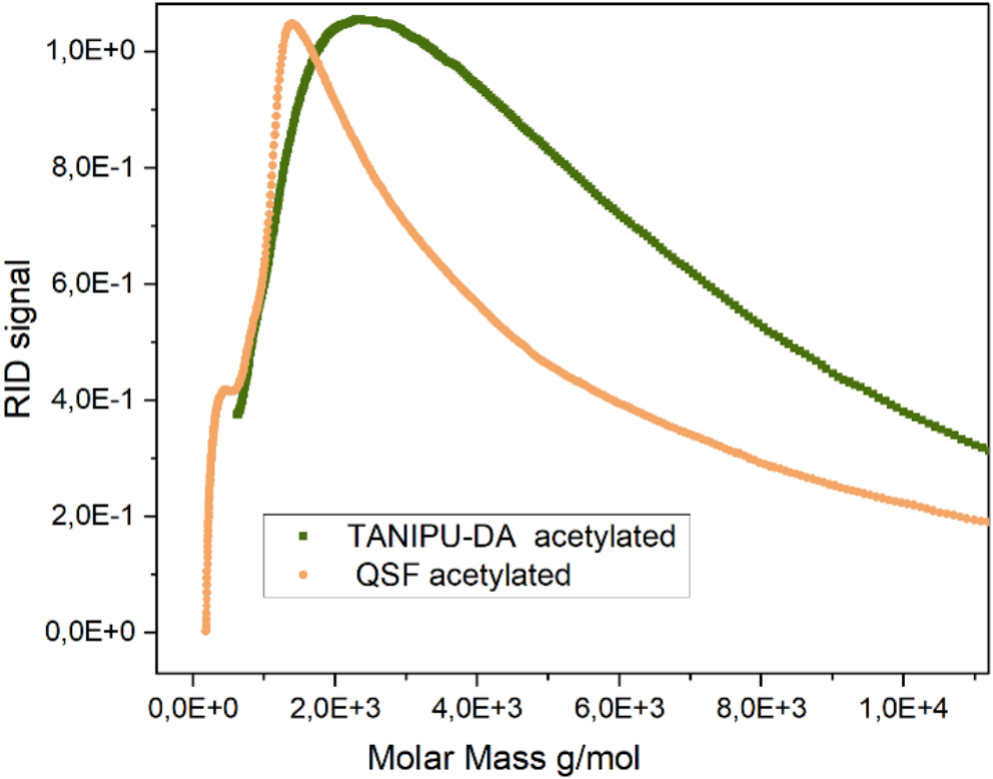
SEC chromatograms of acetylated, QSF and TANIPU-DA resins reveal an increase in molecular size upon stage B resin synthesis

### 3.3 Thermal Stability and Cure Characterization of Stage B TANIPU Resins

The thermogravimetric analysis of QSF, QSF-Cb, and TANIPU resins provided insights into their thermal stabilities and compositions. Four primary peaks of thermal degradation are noted (Figure 5 and Table 2). The initial mass loss, seen in all four samples (<8%), occurs between 25–150 °C and is probably due to the evaporation of water and remaining solvent. Approximately at 180 °C, a second peak in thermal degradation is identified for QSF-Cb, featuring a mass loss of about 12%. It stems from the release of carbonate groups, as confirmed by TGA/MS, where peaks at *m*/*z* = 15, 18, 31, 44, 59, and 90 Da were identified (Figure S3). The prominent degradation corresponding to 40% mass loss for QSF and QSF-Cb occurs at around 279 and 265 °C, respectively, suggesting that QSF-Cb is less stable than QSF (Table 2).

**Figure 5:**
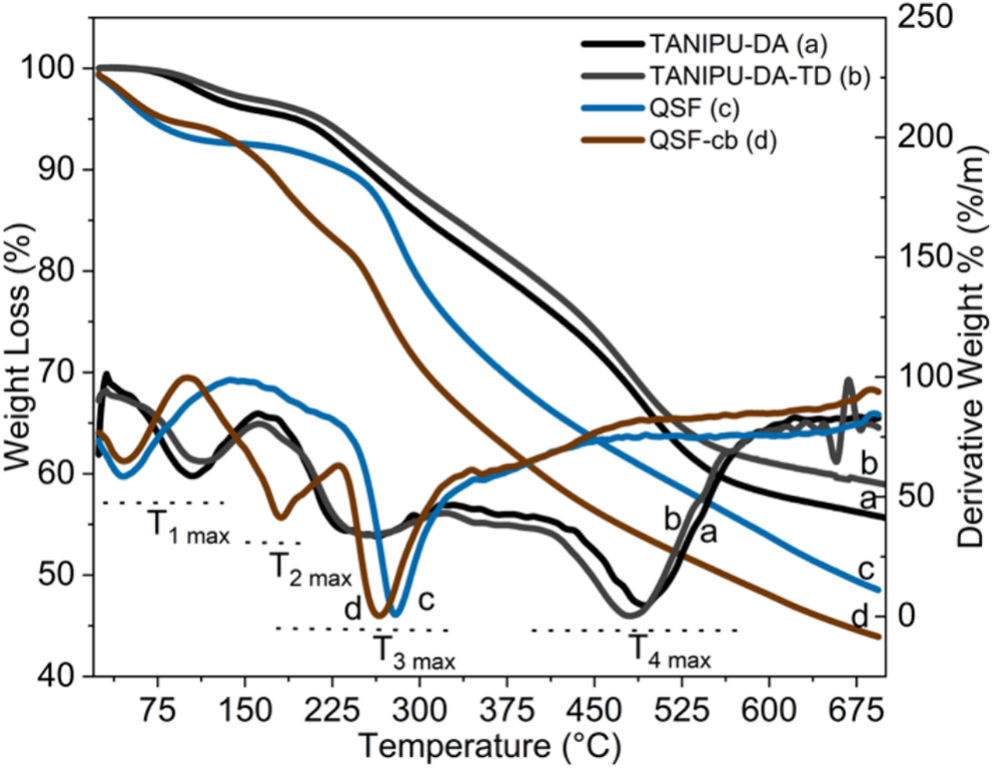
TGA curves of QSF, QSF-Cb, TANIPU-DA and TANIPU-DA-TD resins.

**Table 2:** Thermal Stability during Various Stages of Degradation of QSF, QSF-Cb and Stage B-resins; TANIPU-DA and TANIPU-DA-TD

TGA and DTG	residual weight (%) at 700 °C (W_700_ °C)	maximum temperature (°C) of degradation (T_1 max_) and associated weight loss (%)	maximum temperature (°C) of degradation (T_2 max_) and associated weight loss (%)	maximum temperature (°C) of degradation (T_3 max)_ and associated weight loss (%)	maximum temperature (°C) of degradation (T_4 max)_ and associated weight loss (%)
sample	W_700_ °C	T_1 max_	T_2 max_	T_3 max_	T_4 max_
QSF	48	45 (7.3%)		279 (44%)	
QSF-Cb	44	45 (5.5%)	180 (12%)	265 (39%)	
TANIPU-DA	56	104 (4%)		263 (13.5%)	493 (26.5%)
TANIPU-DA-TD	59	109 (3%)		256 (13%)	480 (26%)

This third degradation peak (T_3 max_,) is also observed in TANIPU-DA and TANIPU-DA-TD, albeit representing only a 13% mass loss. This peak is likely the signature for the thermal degradation of QSF and QSF-Cb, suggesting that there is only approximately 13% of unreacted or carbonated condensed tannins. Alternatively, the TANIPU-DA and TANIPU-DA-TD resins experience a fourth prominent degradation peak (T_4 max_) at 480 and 496 °C, respectively, which accounts for approximately 26% of their mass loss. Indeed, summing the mass losses associated with the third and fourth degradation peaks in both TANIPU resins, a total mass loss of 39% is obtained, which corresponds to that observed for QSF and QSF-Cb in the third peak. The fourth thermal degradation peak of TANIPU resins thus likely refers to the degradation of condensed NIPU chains.

The curing characterization of Stage B TANIPU resins was conducted using DMA. The analysis reveals that the storage modulus (*E*′) and loss modulus (*E*″) increase from approximately 130 °C to about 220 °C in a two-stage process (Figure 6a). Initially, a sharp rise in *E*′ occurs from a minimum plateau (*E*′_min_) to an intermediate plateau (*E*′_mid_) at around 130 °C, which coincides with a loss modulus peak and is thus attributed to gelation. The second sharp increase occurs in the temperature range from 160 to 230 °C, with an inflection point in *E*′ noted around 190 °C and associated again with the *E*″ peak, indicating vitrification. The corresponding *E*″ peaks confirm the assignments to high energy dissipation processes, ascribed to gelation and vitrification. The evolution of *E*′ as a function of time can be recomputed into a mechanical degree of cure as.^45,46^

$$\mathrm{mechanical degree}\;\left(ß\left(t\right)\right)=\frac{E^{\prime }\left(t\right)-E^{\prime }\left(0\right)}{E^{\prime }\left(\infty \right)-E^{\prime }\left(0\right)}$$

where *E*′ (0) denotes the onset storage modulus, *E*′ (*t*) is the storage modulus at a time ‘*t*’ during cure and *E*′ (∞) is the final storage modulus at the complete cure plateau. It reveals that TANIPU-DA-TD cures faster than TANIPU-DA and undergoes gelation and vitrification sooner in the thermogram than TANIPU-DA (Figure 6b). This is consistent with the higher average functionality of the DA-TD cross-linking system.

**Figure 6:**
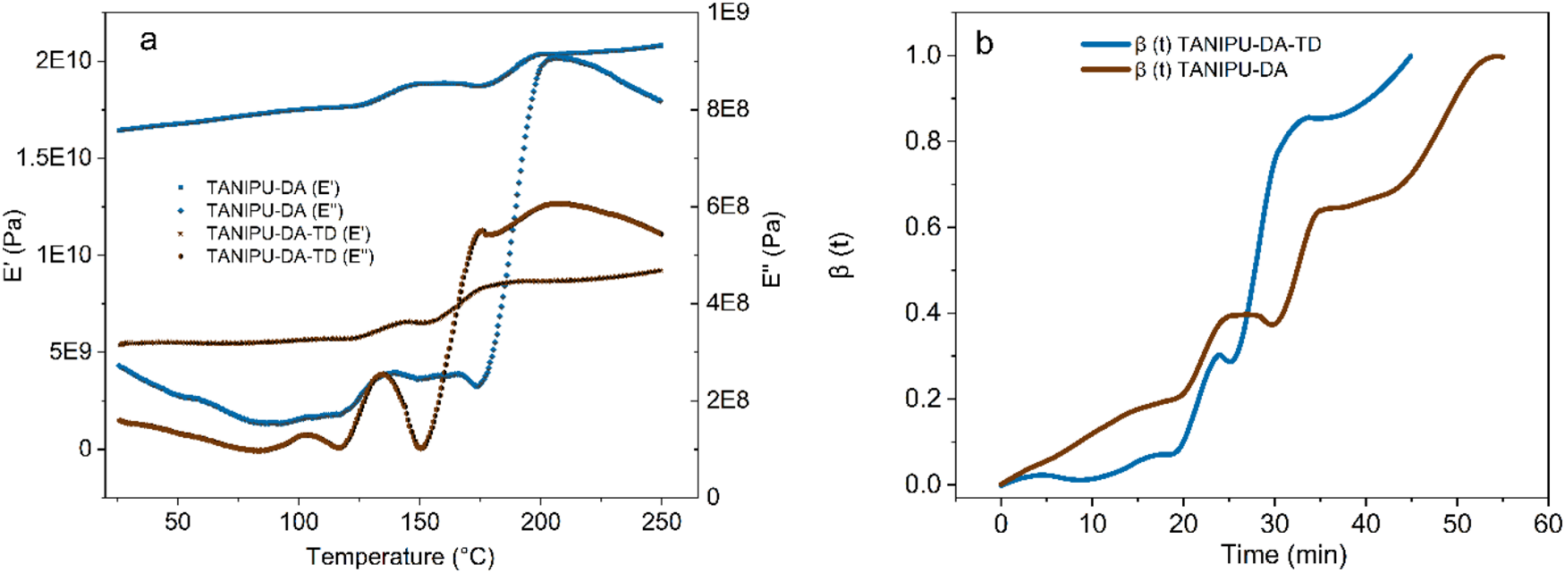
DMA cure thermogram of TANIPU-DA and TANIPU-DA-TD resins during a heating scan at 5 °C/min in single cantilever mode at 1 Hz (a); DMA cure thermogram of TANIPU-DA and TANIPU-DA-TD: The degree of cure as a function of cure time computed from *E*′ in the cure thermogram (b).

Also note that TANIPU-DA-TD achieves complete cure in 45 min, while TANIPU-DA requires 54 min to reach complete cure. This difference in curing kinetics stems from the additional cross-links formed through TD in TANIPU-DA-TD. While the two resins exhibit dynamic moduli of different orders of magnitude, it is worth remembering that pocket powder in DMA is recommended solely for relaxations and is unreliable for moduli values. As the DMA thermograms identified the maximum cure rate between 180 and 220 °C, the isothermal kinetics for network formation in this temperature range were further assessed. Namely, the resins were oven-cured at 200 °C for 5 to 30 min, and their gel content was measured. Oven curing at 200 °C revealed distinct isothermal network formation kinetics for the TANIPU resins (Figure S2). The TANIPU-DA formulation needs at least 30 min of curing at 200 °C to reach its maximum gel content of around 75%. In contrast, TANIPU-DA-TD attains the same gel content in less than 10 min. After 30 min, TANIPU-DA-TD achieves a maximum gel content of 85%.

### 3.4 Shear Bond Strength

An iterative process was conducted to measure the gel time and the hot-pressing of bonded assemblies at temperatures exceeding the gelation point. The bonding strength was evaluated during this procedure per DIN EN 302–1 (2013). For both TANIPU resins, the optimal curing temperature of 200 °C yielded the highest bonding strengths (Figure 7a). This is consistent with the DMA cure characterization, suggesting that vitrification and complete curing occur between 160 and 220 °C. Furthermore, it illustrates that TANIPU-DA-TD consistently exceeds TANIPU-DA in bond strength, irrespective of the curing temperature. When hot-pressed at 200 °C for 10 min, TANIPU-DA-TD achieved a dry strength of 10.12 ± 0.52 N/mm^2^ with 90–100% wood failure (Figure S4), meeting the A1 wood adhesive standard (10 N/mm^2^), while TANIPU-DA closely followed at 9.7 ± 0.5 N/mm^2^ (Figure 7a).

**Figure 7:**
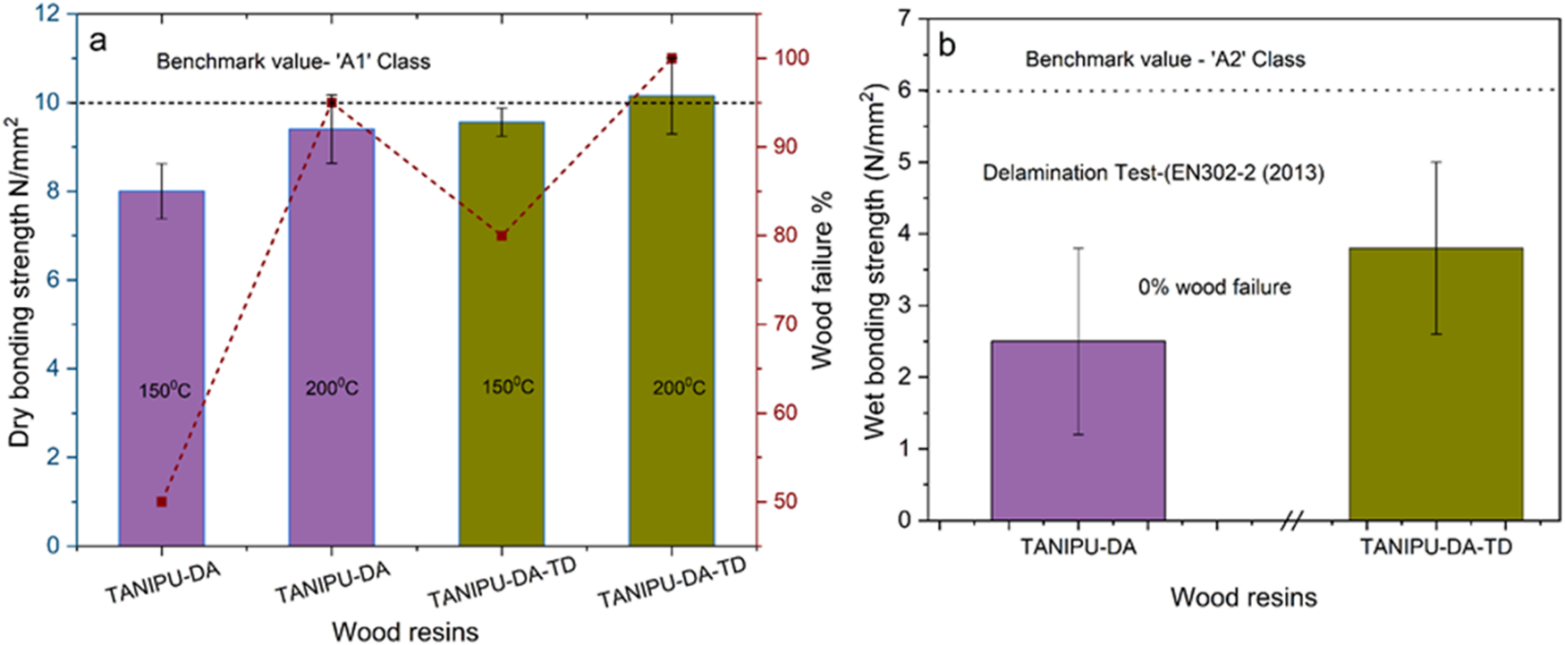
Dry lap shear strength (EN 302–1) of beech wood panel assemblies pressed for 600 s. under a pressure of 1 N/mm^2^ at temperatures of 150 and 200 °C (a) and the lap shear strength in wet conditions (Delamination test, EN 302–2) following the pressing of the beech wood panel assemblies for 600 s. under a pressure of 1 N/mm^2^ pressure and a temperature of 200 °C (b).

While the resin formulations are devoid of isocyanates or formaldehyde, it must be noted that TD can, in principle, decompose into formaldehyde,^47^ albeit, in the presence of highly reactive nucleophilic compounds such as condensed tannins, the released formaldehyde is likely to be captured into the polyflavonoid ring.^48–56^ Additionally, the TANIPU-DA-TD resin is formulated in a high-pH water-based solution (pH 11–12), favoring the formation of highly reactive amino-imine intermediates from TD instead of formaldehyde.^48–51,57,58^ Although these conditions favor the production of formaldehyde-free TANIPU joints, emissions have not been measured. The delamination resistance of TANIPU resins was also assessed in accordance with DIN EN 302–2 (2013) (Figure 7b). TANIPU-DA-TD maintains the best performance at approximately 3.8 ± 1.2 N/mm^2^ in wet adhesion; however, its wet strength is below 6 N/mm^2^, which is the criterion to qualify as an ‘A2’ Class wood adhesive. To our knowledge, this research represents a significant advancement in the field of wood adhesives, as no tannin/lignin-based NIPU wood adhesives have previously been reported^17–19,59–69^ that meets the required dry strength for internal structural applications. The comparison of the performance of TANIPU wood adhesives against traditional wood adhesives (Table 3) emphasizes the significance of this study in the development of more sustainable adhesives, offering a viable, biobased alternative to conventional synthetic wood adhesives that depend on formaldehyde and isocyanates.

**Table 3:** Comparison of the Performance of Traditional Wood Adhesives and Tannin-based Adhesives Documented in Prior Research Publications with This Study Based on the Lap Shear Test

resin	substrate	bonding strength (MPa)	pressed parameters (dry conditions)	refs
tannin (9%)-HBP (91%)	beech wood	8–9	180 °C, 10 min, 1.5 N/mm^2^ EN 205	^70^
pine tannin-hexamine 6%	beech wood	12–14	150 °C, 15 min, 1.2 N/mm^2^ EN 205	^71^
phenol-formaldehyde	beech wood	9	180 °C, 10 min, 1.5 N/mm^2^, EN 205	^70^
PVAc (Multibond SK8)	beech wood	9.5–17	20 °C, 60 min, 1.2 N/mm^2^ EN 205	^72^
urea-formaldehyde (Rakoll Isarit E1)	beech wood	4.6–14.4	90 °C, 800 s, 0.6 N/mm^2^ EN 205	^73^
3 M Scotch-Weld PUR adhesives	wood	10	24 °C, 2 h	^74^
this study	beech wood	9–10.65	200 °C, 10 min, 1 N/mm^2^ EN 205	TANIPU

In this study, QSF-based NIPUs were synthesized following the established routes of carbonation with DMC and urethanization of carbonate groups with diamines, delivering TANIPU-DA. Additionally, the second cross-linker, TD, served as a source of highly reactive amino-imino methylene bases in the presence of QSF under alkaline pH. It was incorporated into the TANIPU-DA resin formulations, yielding TANIPU-DA-TD resin. The structural characterization of the TANIPU resins provided insights into the chemistry associated with the synthesis of NIPU, from the raw material to stage B resin. The TD cross-linker notably improved the cure kinetics of the resin compared to standard TANIPU-DA; however, a high cure temperature of 200 °C and a relatively extended cure time of around 20 min remained necessary. The bonding performance evaluated according to DIN EN 302–1 revealed closely aligned shear strength values for the resins. The inclusion of TD enabled QSF-based NIPU to reach the A1 class standard of 10 N/mm^2^, fulfilling the requirements for wood composites as specified in DIN-EN 302–1, which has not been previously reported for tannin-based nonisocyanate polyurethane wood resins. Ongoing efforts are focused on enhancing adhesive performance, scaling up production, and validating the TANIPU formulation for load-bearing applications. Additionally, these efforts aim to diversify the formulation portfolio at the laboratory scale, thereby expanding the range of potential adhesive applications.

## Supplementary Material



## Data Availability

The data sets used and/or analyzed during the current study will be available upon request.

## Notes

MALDI mass Spectrum of quebracho tannin, Figure- isothermal oven cure on the gel content of the stage B, TANIPU resins, TGA/MS procedure, Figures-thermal analysis and the MS measurement of QSF-Cb, visual images of wood failure of Lap joined samples from dry and wet bonding tests of TANIPU resins (PDF)

## Abbreviations

CT: condensed tannins
DA: hexamethylene diamine
DBU: 1,8-diazabicyclo­[5.4.0]­undec-7-ene
DMAc: dimethyl acetamide
DMA: dynamic mechanical analysis
DMC: dimethyl carbonate
DS: degree of substitution
DTG: differential thermogravimetric analysis
2D NMR: two-dimensional nuclear magnetic resonance
FTIR: Fourier-transform infrared spectroscopy
HSQC: heteronuclear single quantum coherence
MALDI-TOF: matrix-assisted laser desorption ionization time-of-flight
MS: mass spectrometry
N,N-DMF: *N*,*N*-dimethyl formamide
NIPU: nonisocyanate polyurethane
PMMA: poly­(methyl methacrylate)
^**31**^ **P NMR**: phosphorus-31 nuclear magnetic resonance
QSF Cb: carbonated quebracho tannin extract soluble fraction
QSF: quebracho tannin extract soluble fraction
SEC: size exclusion chromatography
TANIPU: tannin based nonisocyanate polyurethane
TD: hexamethylenetetramine
TGA: thermogravimetric analysis
TMDP: 2-chloro-4,4,5,5-tetramethyl-1,3,2-dioxaphospholate
VOC: volatile organic compounds
