# Supplementary material for: Tuning Polyphenols for Thermosetting Wood Adhesives: Class ‘A1’ Non-Isocyanate Polyurethanes from Condensed Tannins
Source: J Agric Food Chem. 2025 Jun 27;73(27):17057–68. doi: 10.1021/acs.jafc.5c02453 (PMC12257505; doi:10.1021/acs.jafc.5c02453)
Supplement: Supplementary file 1 [file jf5c02453_si_001.pdf]

Manuscript ID jf-2025-02453e_Supporting Information for Publication

Tuning Polyphenols for Thermosetting Wood Adhesives: Class 'A1' Non-Isocyanate Polyurethanes from Condensed Tannins

Gopakumar Sivasankarapillai [a,b], Arsène Bikoro Bi Athomo [a,b], Detlef Schmiedl [c], Antonio Pizzi [d], Marie-Pierre Laborie [a,b, e *]

[a] Freiburg Material Research Centre, University of Freiburg, Stefan-Meier-Strasse 21, D-79104 Freiburg i. Br., Germany
[b] Chair of Forest Biomaterials, Faculty of Environment and Natural Resources, University of Freiburg, Werthmannstrasse 6, D-79085 Freiburg i. Br., Germany
[c] Department of Environmental Engineering, Fraunhofer Institute for Chemical Technology ICT, Joseph-Von-Fraunhofer-Str. 7, 76327, Pfinztal, Germany

[d] Lermab-Enstib, University of Lorraine, 27 rue Philippe Seguin, 88000 Epinal, France.

[e]Institut Charles Sadron, 23 rue du Loess, BP 84047, 67034 STRASBOURG Cedex 2 - France

gopakumar.sivasankarapillai@fmf.uni-freiburg.de (G.S.); arsene.bikoro@biomat.uni-freiburg.de (A.B.B.A.); detlef.schmiedl@ict.fraunhofer.de (D.S.); antonio.pizzi@univ-lorraine.fr (A.P.); * Correspondence :marie-pierre.laborie@biomat.uni-freiburg.de (M.P.L.)

MALDI mass Spectrum of quebracho tannin extract (QSF)

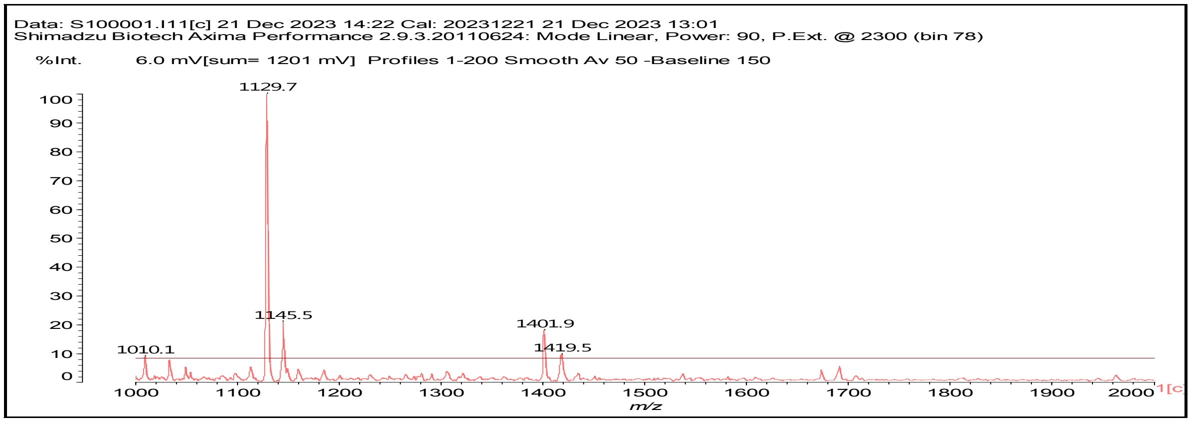

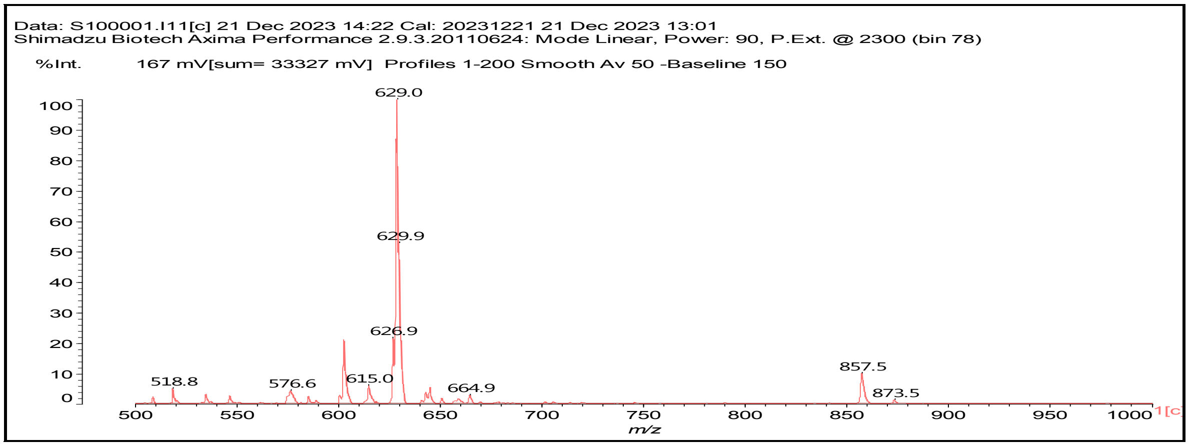

Figure S1: MALDI mass Spectrum of quebracho tannin extract (QSF)

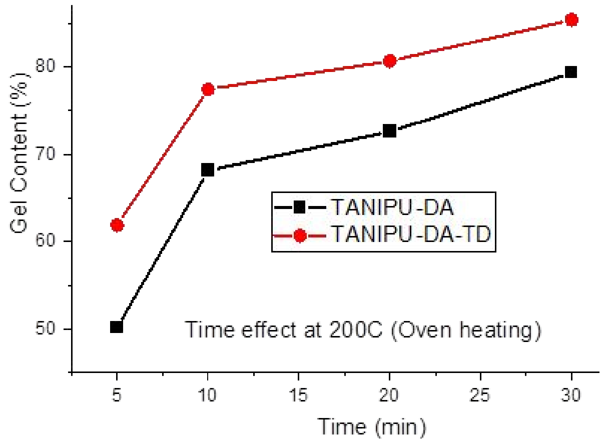

Figure S2: Impact of isothermal oven cure time at 200°C on the gel content of the stage B, TANIPU resins

Thermal analysis and the MS measurement of QSF-Cb

Analysis equipment: TG-MS from Netzsch; TG 209 F1 Iris ASC coupled with QMS 403 C Aeolos, Analytical balance Delta Range XP 26 from Mettler ToledoImplementation:

The decomposition curves of the samples were measured using the vacuum-tight NETZSCH TG 209 F1 Iris thermobalance. This balance can be used to carry out tests in the temperature range between room temperature and 1000°C with a resolution of 0.1µg. Two purge gas inlets and a protective gas are available for the analyses, which are precisely controlled in the device via an integrated mass flow controller. The data acquisition as well as the control and evaluation of the measurements were carried out using Netzsch's Proteus software.

The gases released from the sample at certain temperatures are fed into the measuring cell of the mass spectrometer via a transfer line thermostatted to 280°C. The start of the thermal analysis and the MS measurement is triggered by the software, so that both data can be measured and recorded simultaneously. The MS data was evaluated using Bruker Instruments' Quadstar software.

Approx. 25 mg of the sample was weighed into an aluminum oxide crucible. The crucibles were placed in the thermobalance and the following measuring program was started.

Table S1 Working parameter at the TG

Parameter	Device setting
Temparature range	25°C-200 °C
Gas	N_2
Heating rate	10 K/min
Gas flow	40mL/min
crucible	Al_2O_3

In addition to the signals at mass numbers 18 (H_2O) and 44 (CO_2), the mass spectrometer also showed m/z 15; 31 (weak: 29-33); 42-46; 59 (weak: 58; 60-62) and 90 (weak signal) - these could be assigned to dimethyl carbonate. Only m/z 18 appears in the temperature range between 40 - 120°C. For the remaining m/z_s, an increase in signal intensity is visible from 100°C.

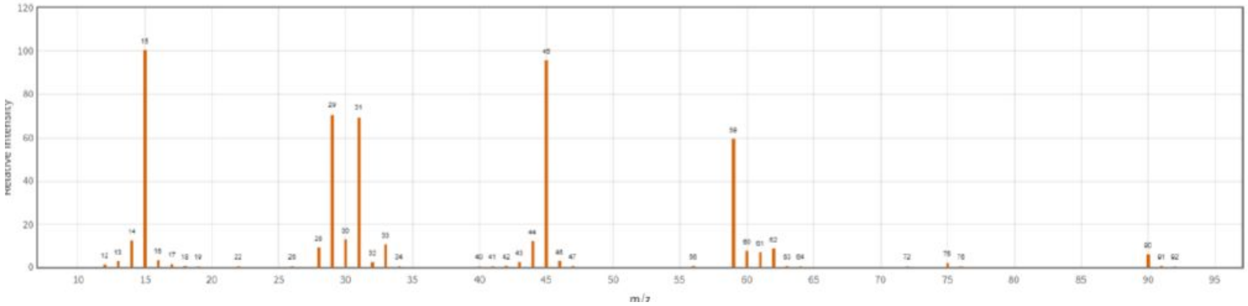

Figure S3: Mass spectrum of Dimethyl carbonate

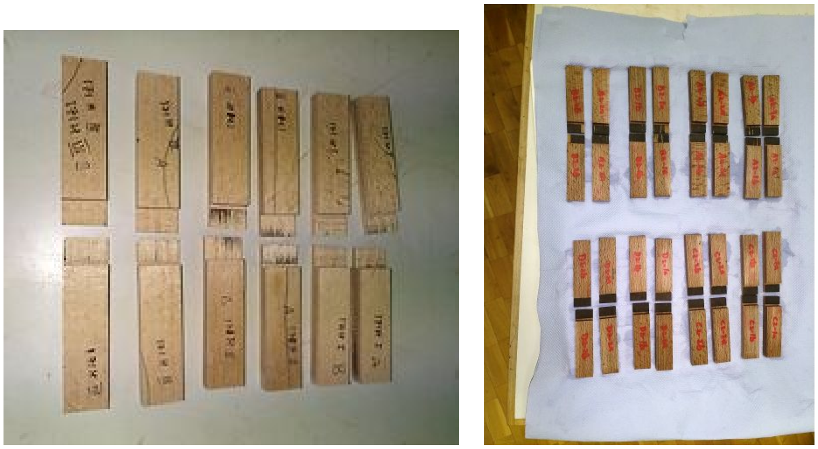

Figure S4 : Visual images of wood failure of Lap joined samples from (a) dry and b) wet bonding strength tests of TANIPU resins, according to EN 302-(1&2) standard methods
